# Mislabeling and inappropriate analysis render interpretations from “The effect of the participatory heat education and awareness tools (HEAT) intervention on agricultural worker physiological heat strain: results from a parallel, comparison, group randomized study” unreliable

**DOI:** 10.1186/s12889-026-26681-1

**Published:** 2026-04-30

**Authors:** Chelsi M. Webster, Yasaman Jamshidi-Naeini, Lilian Golzarri-Arroyo, Colby J. Vorland, Zachary J. Schlader, Blair D. Johnson, David B. Allison

**Affiliations:** 1 Department of Epidemiology and Biostatistics, Indiana University School of Public Health-Bloomington, 1025 E 7th St, PH 111, Bloomington, IN 47405 USA; 2 Department of Kinesiology, Indiana University School of Public Health-Bloomington, Bloomington, IN USA; 3 USDA Children’s Nutrition Research Center at Baylor College of Medicine, Department of Pediatrics, and Texas Children’s Hospital, 1100 Bates St., Houston, TX 77030 USA

**Keywords:** Agricultural workers, Core body temperature, Degrees of freedom, Heat-related illness, Heat stress, Intervention study, Nonrandom assignment, Random allocation, Randomization approaches, Re-analysis

Chavez Santos and colleagues conducted a novel study to test the effectiveness of participatory heat education and a decision-support mobile application on heat strain in farmworkers [1], an important topic to protect an increasingly vulnerable group. The study is presented as a group randomized trial (such trials are often referred to as cluster-randomized trials (cRCTs)). However, not all participants/groups were allocated randomly. In addition, the potential lack of independence of observations due to allocation of groups (rather than individual participants) to study conditions was not accounted for in statistical analyses. These features mean that the statistical analysis is incorrect and the description of the study as a randomized trial is false. Corrections are warranted.

First, the trial is mislabeled as a randomized trial because of the employed randomization approaches. In brief, the study involved three companies, which were originally four companies, two of which were considered as one. Each company was made up of two crews, resulting in a total of six crews. The size of each crew ranged from 8 to 17 individuals. According to the original publications [1, 2], the allocation of participating crews in two of the companies, referred to as “Large 1” and “Large 2” in the article, was determined by a single coin flip. This process ensured that one crew per company was assigned to each study condition, with randomization stratified by company. However, for the third company (referred to as “Small”), the allocation of study conditions was not randomized. Instead, the order of crew enrollment determined allocation: the first enrolled crew received intervention, while the second crew was assigned to the comparison group. The choice to use mixed random and nonrandom assignment means not all participants had a known probability of being assigned to intervention or comparison groups. In essence, some participants had 0% probability of being assigned to the condition previously filled. We previously wrote on aspects of nonrandom, random, restricted, and group random methods [3, 4]. Further, as noted by an anonymous reviewer, “Assuming the allocation of the third company’s work crews was deterministic, then there are only four possible random allocations in the study (two in company 1 times two in company 2). Thinking in terms of these permutations that would imply there are only four possible p-values, over the randomisation distribution. This further undermines the claim of experimental inference.”

Second, randomization occurred at the crew level, and when subjects are randomized in groups of two or more versus individually, this is denoted as group-randomized or cluster-randomized [5]. In such cases, model residuals are (usually positively) correlated (i.e., not independently and identically distributed [5]), and this should be accounted for in analyses by adjusting the degrees of freedom for the number of randomized clusters [6]. The current analysis includes individual “workers” as random effects in linear mixed effects models to account for repeated measures (longitudinal data), and the fully adjusted Model 2 includes the covariate of company as a stratification variable as is required in stratified randomization [1]. Still, clustering (i.e., potential correlation among model residuals) and nesting (i.e., limited degrees of freedom) were not accounted for properly. We contacted the authors to inquire about their analysis approach, and they confirmed that they did not account for clustering and nesting in their analyses. Correct analysis for the experimental design would account for crews as a random effect in all analyses and adjust degrees of freedom for the number of crews [4]. Because the employed approaches disregard extra variation and limited degrees of freedom, the analyses potentially (and likely) have magnified type I error rates [7], calling into question the validity of the presented statistical results and thus the interpretation of the data.

We requested de-identified raw data from the authors to conduct a re-analysis of the published results using appropriate statistical methods for the study design. Although it is stated in the article that “de-identified data and statistical code will be made available […] on reasonable request,” de-identified raw data were not provided to us. In further correspondence, the authors obligingly conducted and shared re-analyzed results for Table 3 of their original article purportedly including a random effect for crew with individuals nested within crew for Models 1 and 2 [1]. In the results shared with us, two of the three statistically significant effects originally reported in Model 1 of Table 3 [1] were not statistically significant in the re-analysis model. It is unclear what software was used for re-analysis and whether degrees of freedom were adjusted. The authors did not respond to our inquiries on these matters.

Inconsistency in the randomization methods used in this study undermines some of the assumptions required to draw causal inferences from randomized trials. Therefore, the authors should consider revising their article to clarify to readers that the study is not a randomized controlled trial. Importantly, the interventional design of this trial would be expected to give stronger causal implication than purely observational analyses. We also urge the authors to conduct further re-analyses that account for clustering effects and adjusted degrees of freedom and provide additional details on the model specifications, software used, and other methodological choices made during re-analysis. These revisions are essential to properly interpret the study’s findings. A similar approach was taken in a correction to present re-analyzed results and corrected methods in Vorland et al. [3], which is consistent with the Committee on Publication Ethics (COPE) Retraction Guidelines [8].

With the public health implications at stake for agricultural workers and their increasing risk of heat exposure, it is essential that studies are rigorously and transparently designed and analyzed to produce reliable knowledge. The intervention by Chavez Santos et al. is an important contribution, however, until the necessary revisions are made, the published results and conclusions drawn from this study should not be considered reliable.

## Data Availability

No datasets were generated or analysed during the current study.

## Abbreviations

COPE: Committee on Publication Ethics
cRCT: Cluster randomized controlled trial
HEAT: Heat education and awareness tools
